# Supplementary material for: Tracing the Origin of Planktonic Protists in an Ancient Lake
Source: Microorganisms. 2020 Apr 9;8(4):543. doi: 10.3390/microorganisms8040543 (PMC7232311; doi:10.3390/microorganisms8040543)
Supplement: Supplementary file 1 [file microorganisms-08-00543-s001.zip › Suppl_rev/S_3_alveolates_no ciliates.pdf]

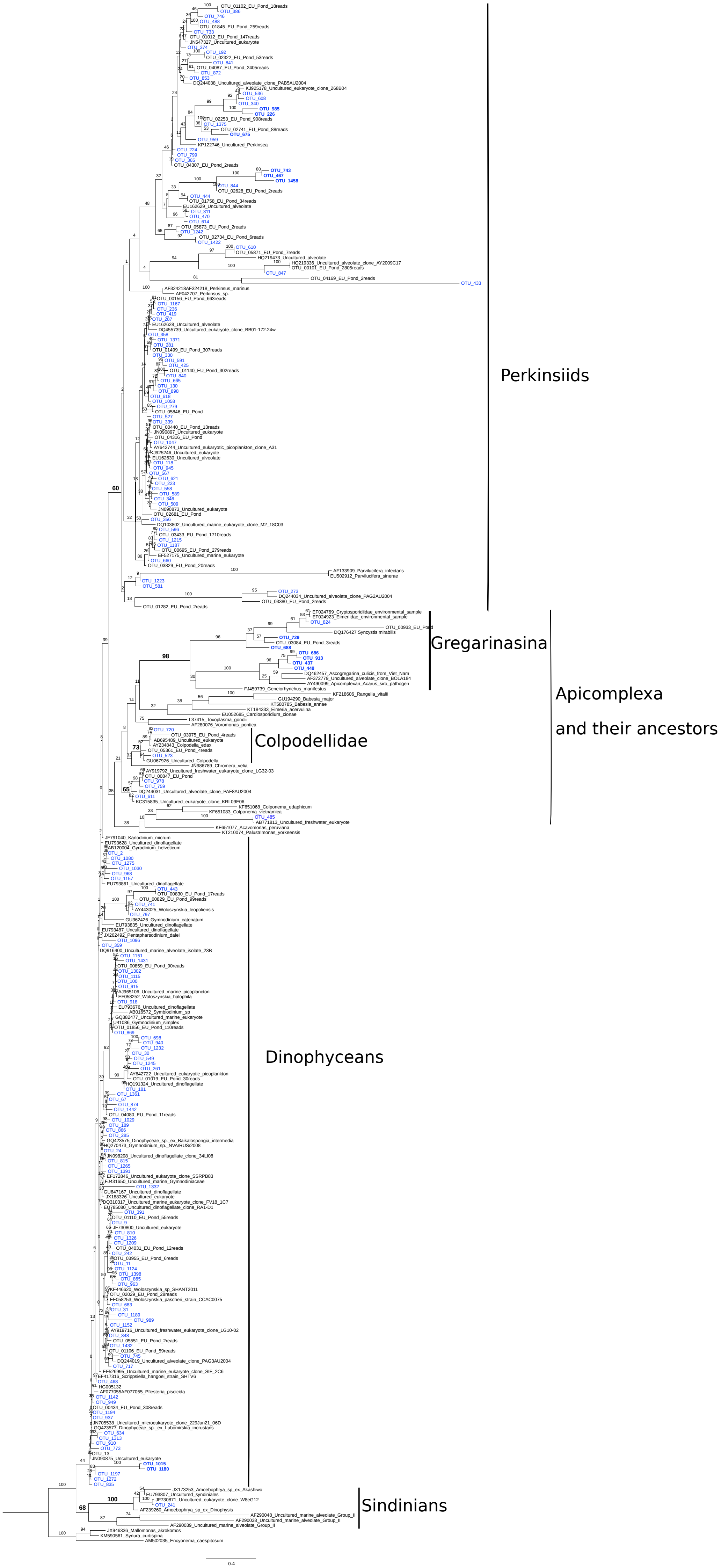
Perkinsiids
Gregarinasina
Apicomplexa
and their ancestors
Colpodellidae
Dinophyceans
Sindinians
0.4
