# Supplementary material for: Tracing the Origin of Planktonic Protists in an Ancient Lake
Source: Microorganisms. 2020 Apr 9;8(4):543. doi: 10.3390/microorganisms8040543 (PMC7232311; doi:10.3390/microorganisms8040543)
Supplement: Supplementary file 1 [file microorganisms-08-00543-s001.zip › Suppl_rev/S_6_Ciliate_.pdf]

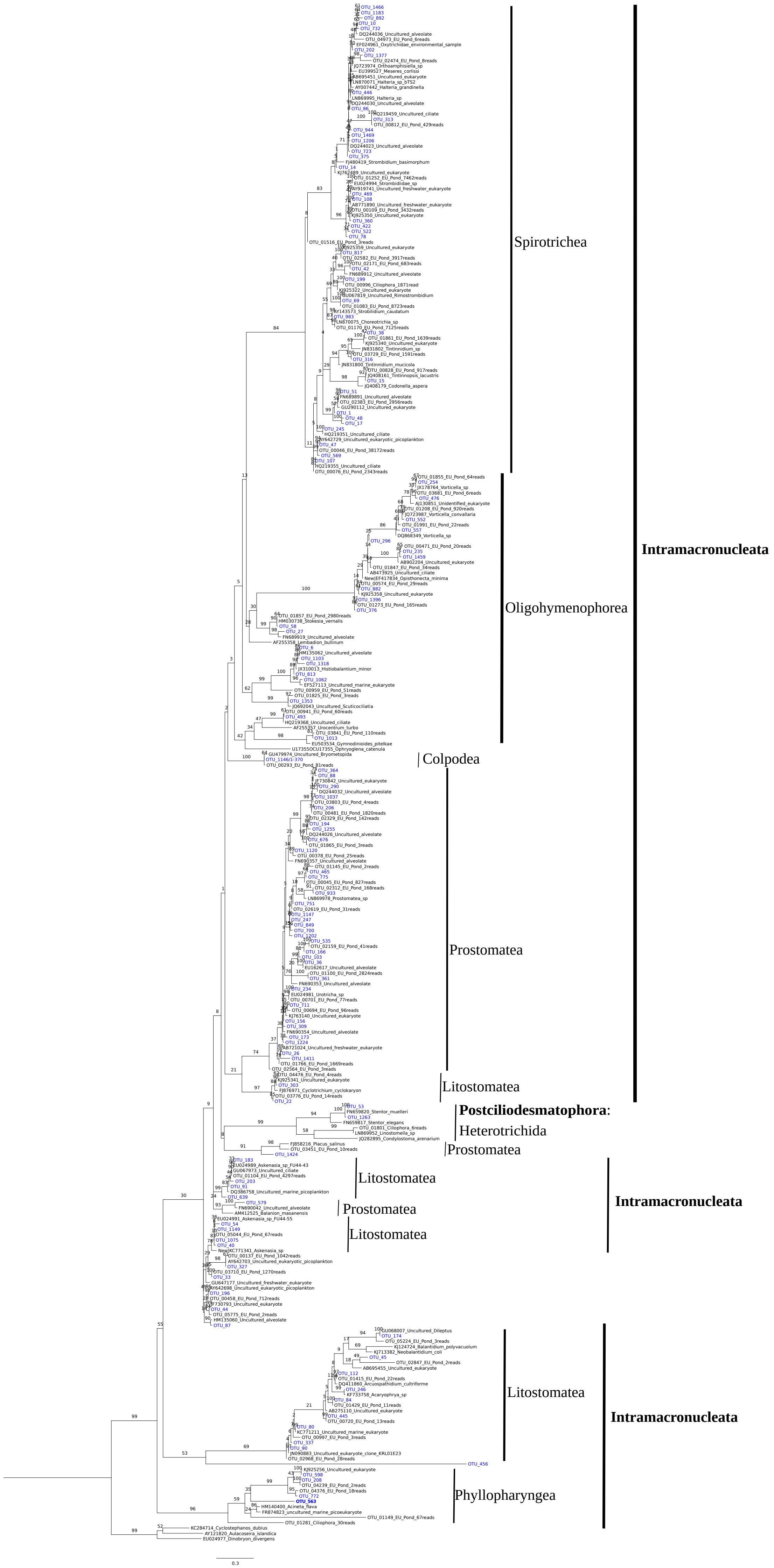
Spirotrichea
Oligohymenophorea
Intramacronucleata
Colpodea
Prostomatea
Litostomatea
Postciliodesmatophora: Heterotrichida
Prostomatea
Litostomatea
Prostomatea
Litostomatea
Intramacronucleata
Litostomatea
Intramacronucleata
Phyllopharyngea
0.3
