# Supplementary material for: Tracing the Origin of Planktonic Protists in an Ancient Lake
Source: Microorganisms. 2020 Apr 9;8(4):543. doi: 10.3390/microorganisms8040543 (PMC7232311; doi:10.3390/microorganisms8040543)
Supplement: Supplementary file 1 [file microorganisms-08-00543-s001.zip › Suppl_rev/S_2_Ochrophyta.pdf]

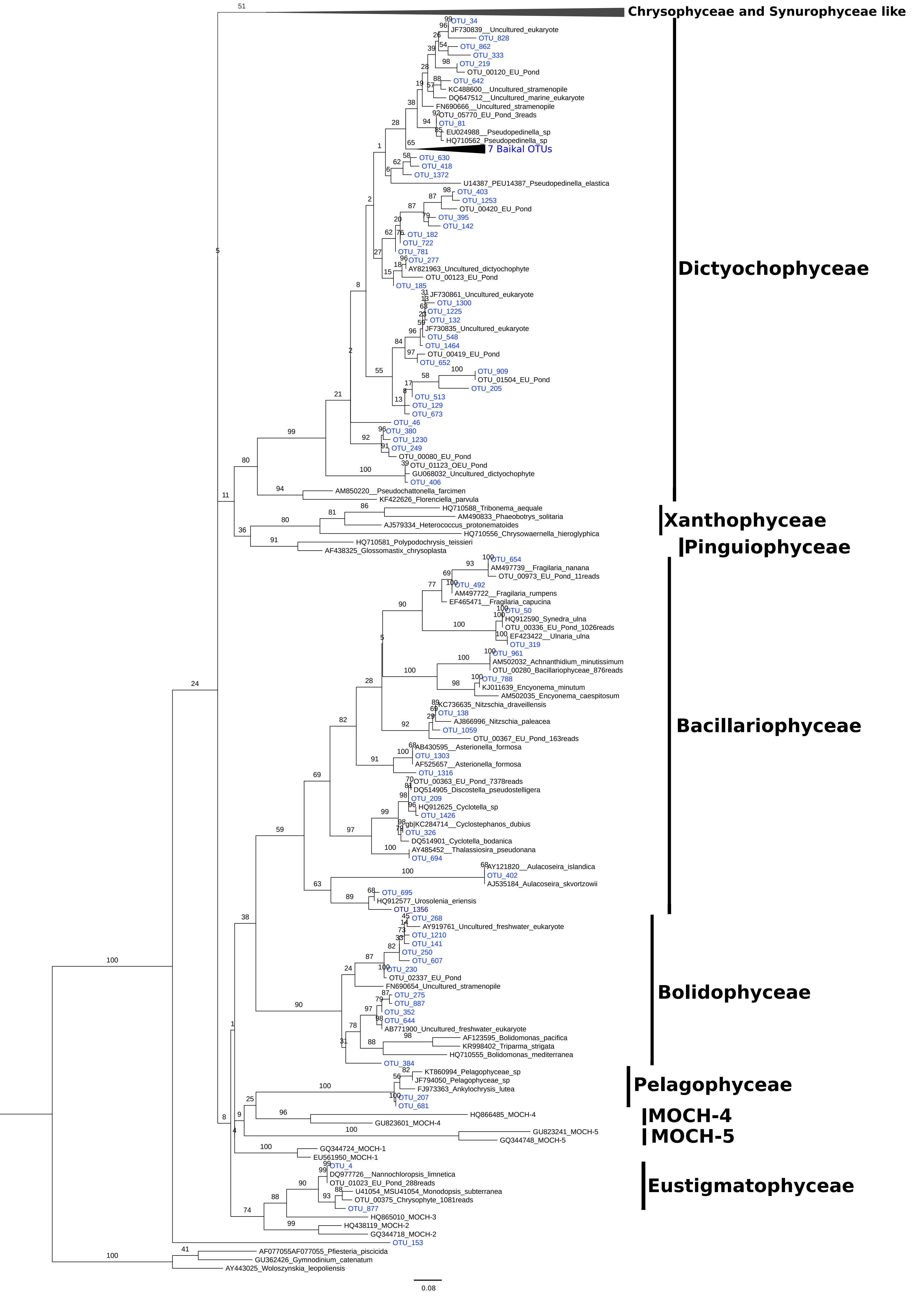
Chrysophyceae and Synurophyceae like
OTU_34
JF730839__Uncultured_eukaryote
OTU_828
OTU_862
OTU_333
OTU_219
OTU_00120_EU_Pond
OTU_642
KC488600__Uncultured_stramenopile
DQ647512__Uncultured_marine_eukaryote
FN690666__Uncultured_stramenopile
OTU_05770_EU_Pond_3reads
OTU_81
EU024988__Pseudopedinella_sp
HQ710562_Pseudopedinella_sp
7 Baikal OTUs
OTU_630
OTU_418
OTU_1372
U14387_PEU14387_Pseudopedinella_elastica
OTU_403
OTU_1253
OTU_00420_EU_Pond
OTU_395
OTU_142
OTU_182
OTU_722
OTU_781
OTU_277
AY821963_Uncultured_dictyochophyte
OTU_00123_EU_Pond
OTU_185
JF730861_Uncultured_eukaryote
OTU_1300
OTU_1225
OTU_132
JF730835_Uncultured_eukaryote
OTU_548
OTU_1464
OTU_00419_EU_Pond
OTU_652
OTU_909
OTU_01504_EU_Pond
OTU_205
OTU_513
OTU_129
OTU_673
OTU_46
OTU_380
OTU_1230
OTU_249
OTU_00080_EU_Pond
OTU_01123_OEU_Pond
GU068032_Uncultured_dictyochophyte
OTU_406
AM850220__Pseudochattonella_farcimen
KF422626_Florenciella_parvula
Dictyochophyceae
HQ710588_Tribonema_aequale
AM490833_Phaeobotrys_solitaria
AJ579334_Heterococcus_protonematoides
HQ710556_Chrysowaernella_hieroglyphica
Xanthophyceae
HQ710581_Polypodochrysis_teissieri
AF438325_Glossomastix_chrysoplasta
Pinguiophyceae
OTU_654
AM497739__Fragilaria_nanana
OTU_00973_EU_Pond_11reads
OTU_492
AM497722__Fragilaria_rumpens
EF465471__Fragilaria_capucina
OTU_50
HQ912590_Synedra_ulna
OTU_00336_EU_Pond_1026reads
EF423422__Ulnaria_ulna
OTU_319
OTU_961
AM502032_Achnanthidium_minutissimum
OTU_00280_Bacillariophyceae_876reads
OTU_788
KJ011639_Encyonema_minutum
AM502035_Encyonema_caespitosum
KC736635_Nitzschia_draveillensis
OTU_138
AJ866996_Nitzschia_paleacea
OTU_1059
OTU_00367_EU_Pond_163reads
AB430595__Asterionella_formosa
OTU_1303
AF525657__Asterionella_formosa
OTU_1316
OTU_00363_EU_Pond_7378reads
DQ514905_Discostella_pseudostelligera
OTU_209
HQ912625_Cyclotella_sp
OTU_1426
gb|KC284714__Cyclostephanos_dubius
OTU_326
DQ514901_Cyclotella_bodanica
AY485452__Thalassiosira_pseudonana
OTU_694
AY121820__Aulacoseira_islandica
OTU_402
AJ535184_Aulacoseira_skvortzowii
OTU_695
HQ912577_Urosolenia_eriensis
OTU_1356
Bacillariophyceae
OTU_268
AY919761_Uncultured_freshwater_eukaryote
OTU_1210
OTU_141
OTU_250
OTU_607
OTU_230
OTU_02337_EU_Pond
FN690654_Uncultured_stramenopile
OTU_275
OTU_887
OTU_352
OTU_644
AB771900_Uncultured_freshwater_eukaryote
AF123595_Bolidomonas_pacifica
KR998402_Triparma_strigata
HQ710555_Bolidomonas_mediterranea
OTU_384
Bolidophyceae
KT860994_Pelagophyceae_sp
JF794050_Pelagophyceae_sp
FJ973363_Ankylochrysis_lutea
OTU_207
OTU_681
Pelagophyceae
HQ866485_MOCH-4
GU823601_MOCH-4
MOCH-4
GU823241_MOCH-5
GQ344748_MOCH-5
MOCH-5
GQ344724_MOCH-1
EU561950_MOCH-1
OTU_4
DQ977726__Nannochloropsis_limnetica
OTU_01023_EU_Pond_288reads
U41054_MSU41054_Monodopsis_subterranea
OTU_00375_Chrysophyte_1081reads
OTU_877
Eustigmatophyceae
HQ865010_MOCH-3
HQ438119_MOCH-2
GQ344718_MOCH-2
OTU_153
AF077055AF077055_Pfiesteria_piscicida
GU362426_Gymnodinium_catenatum
AY443025_Woloszynskia_leopoliensis
0.08
