# Supplementary material for: Tracing the Origin of Planktonic Protists in an Ancient Lake
Source: Microorganisms. 2020 Apr 9;8(4):543. doi: 10.3390/microorganisms8040543 (PMC7232311; doi:10.3390/microorganisms8040543)
Supplement: Supplementary file 1 [file microorganisms-08-00543-s001.zip › Suppl_rev/S7_archaeplastida_.pdf]

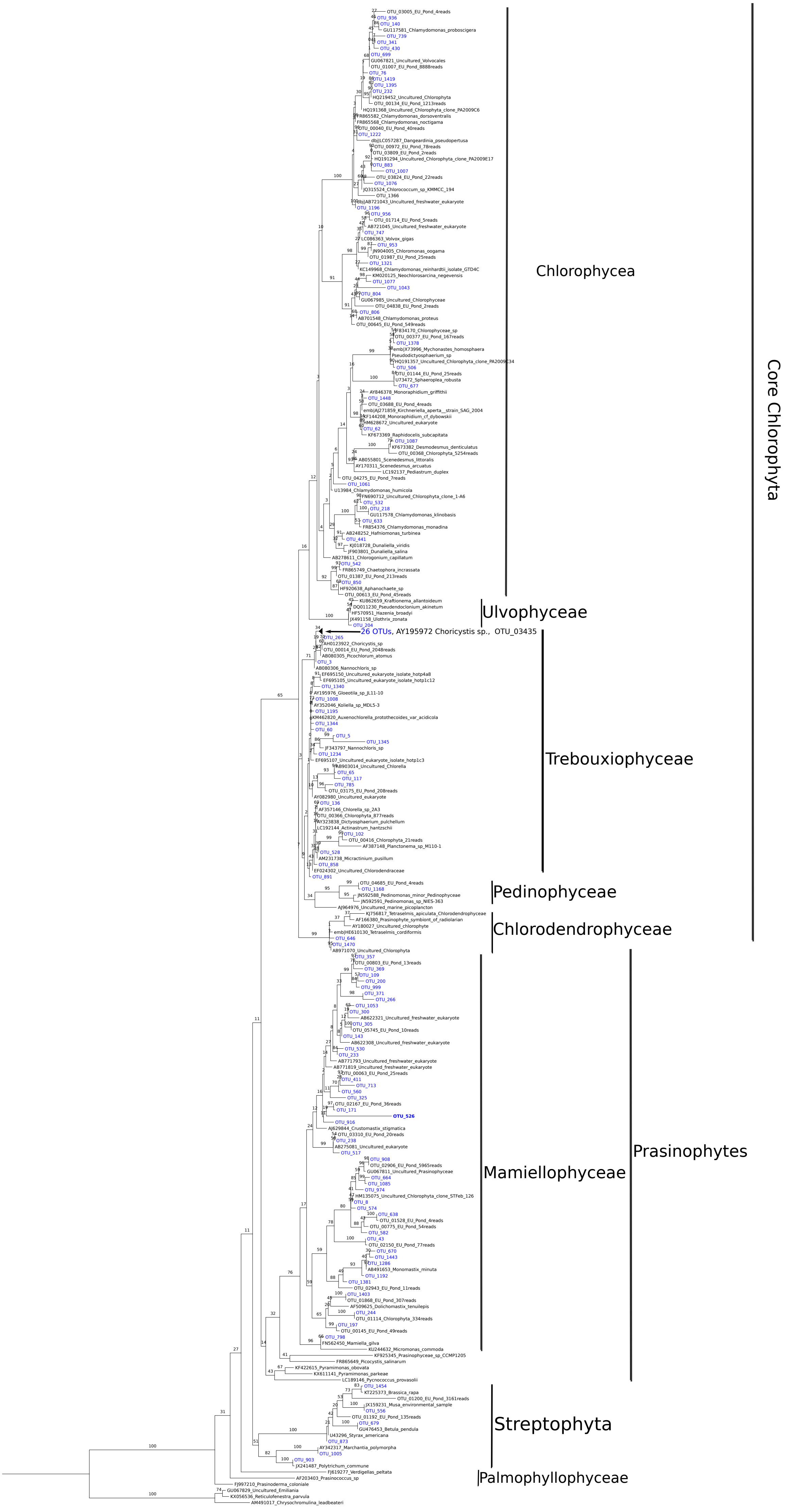

Chlorophycea
Ulvophyceae
26 OTUs, AY195972 Choricystis sp., OTU_03435
Trebouxiophyceae
Pedinophyceae
Chlorodendrophyceae
Mamiellophyceae
Prasinophytes
Streptophyta
Palmophyllophyceae
Core Chlorophyta
