# Supplementary figures and images for: Tracing the Origin of Planktonic Protists in an Ancient Lake
Source: Microorganisms. 2020 Apr 9;8(4):543. doi: 10.3390/microorganisms8040543 (PMC7232311; doi:10.3390/microorganisms8040543)

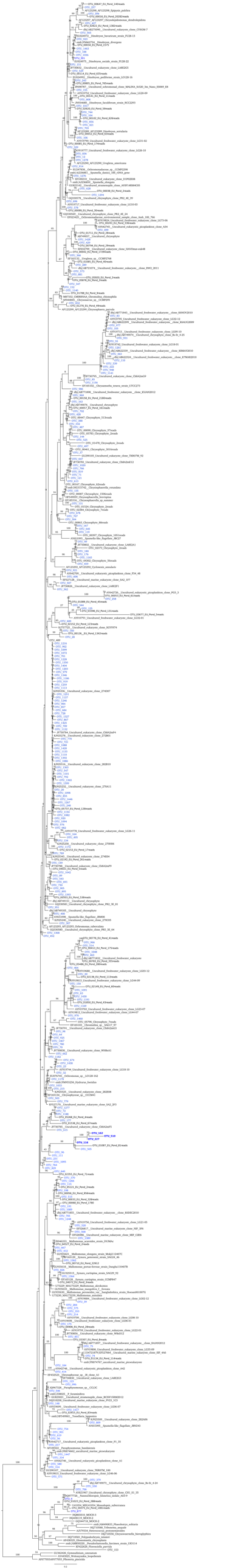

Supplement: Supplementary file 1 [file microorganisms-08-00543-s001.zip › Suppl_rev/S_11_Chrizophyceae.pdf]

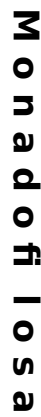

0.4

Supplement: Supplementary file 1 [file microorganisms-08-00543-s001.zip › Suppl_rev/S_5_Rhizaria_.pdf]

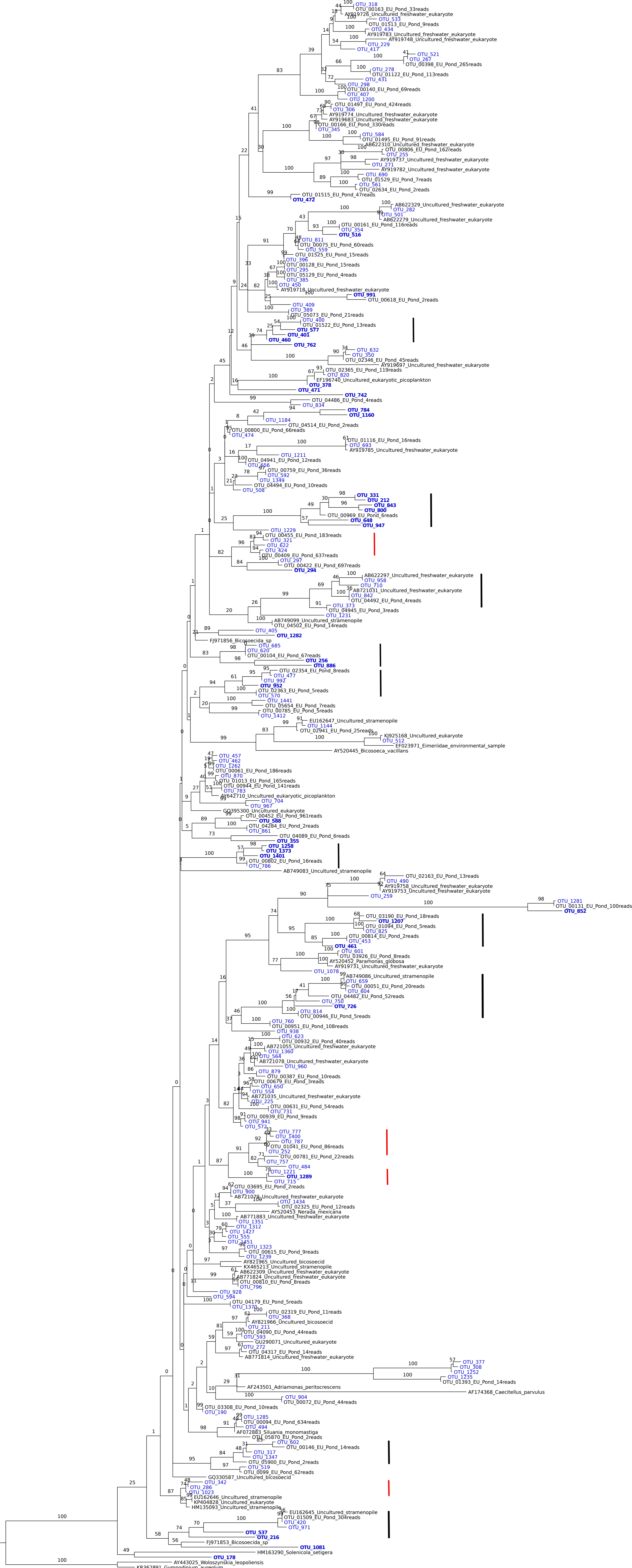

Supplement: Supplementary file 1 [file microorganisms-08-00543-s001.zip › Suppl_rev/S_9_bicosoecids_.pdf]

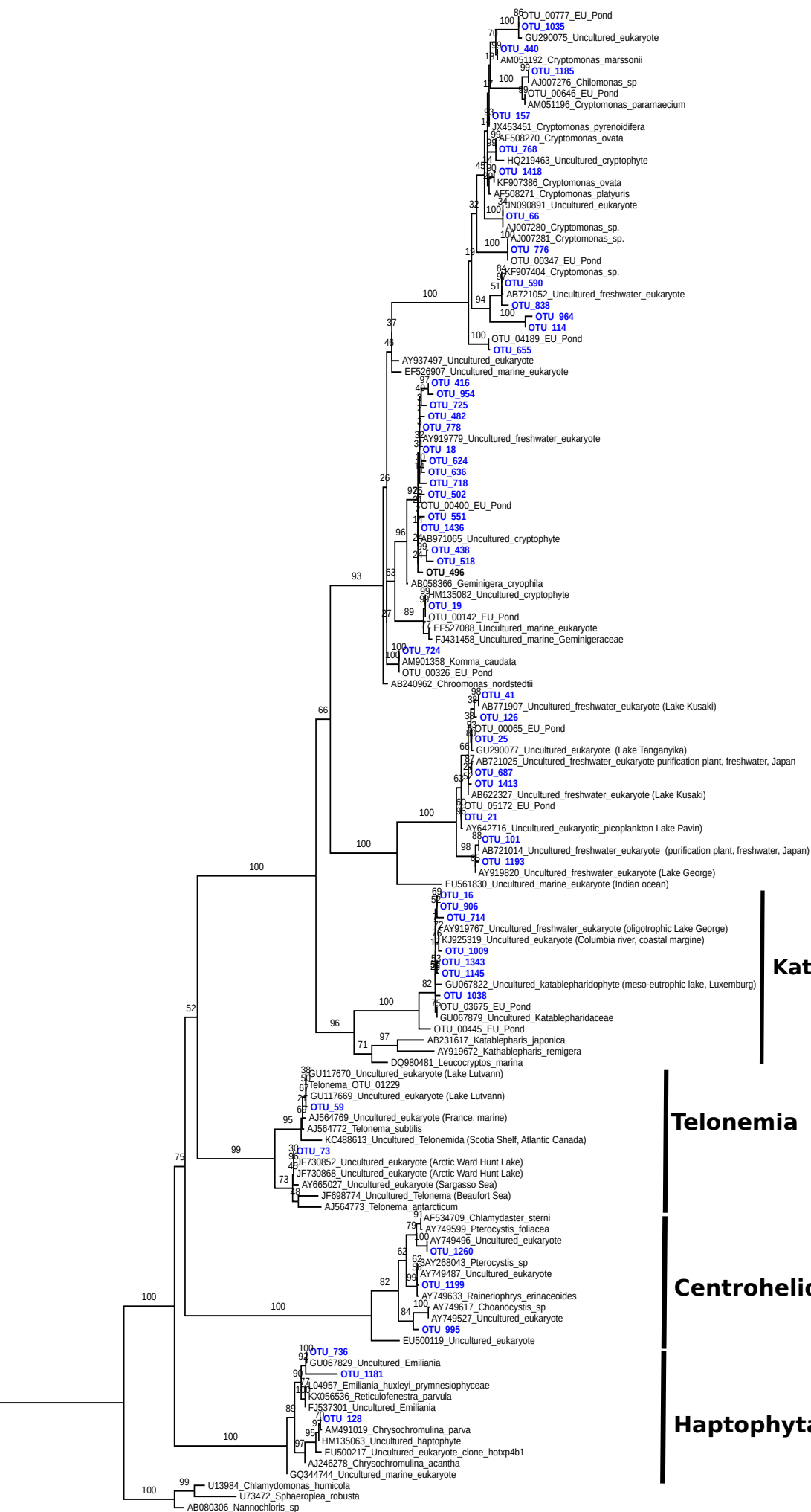

Supplement: Supplementary file 1 [file microorganisms-08-00543-s001.zip › Suppl_rev/S_8_Hacrobia.pdf]

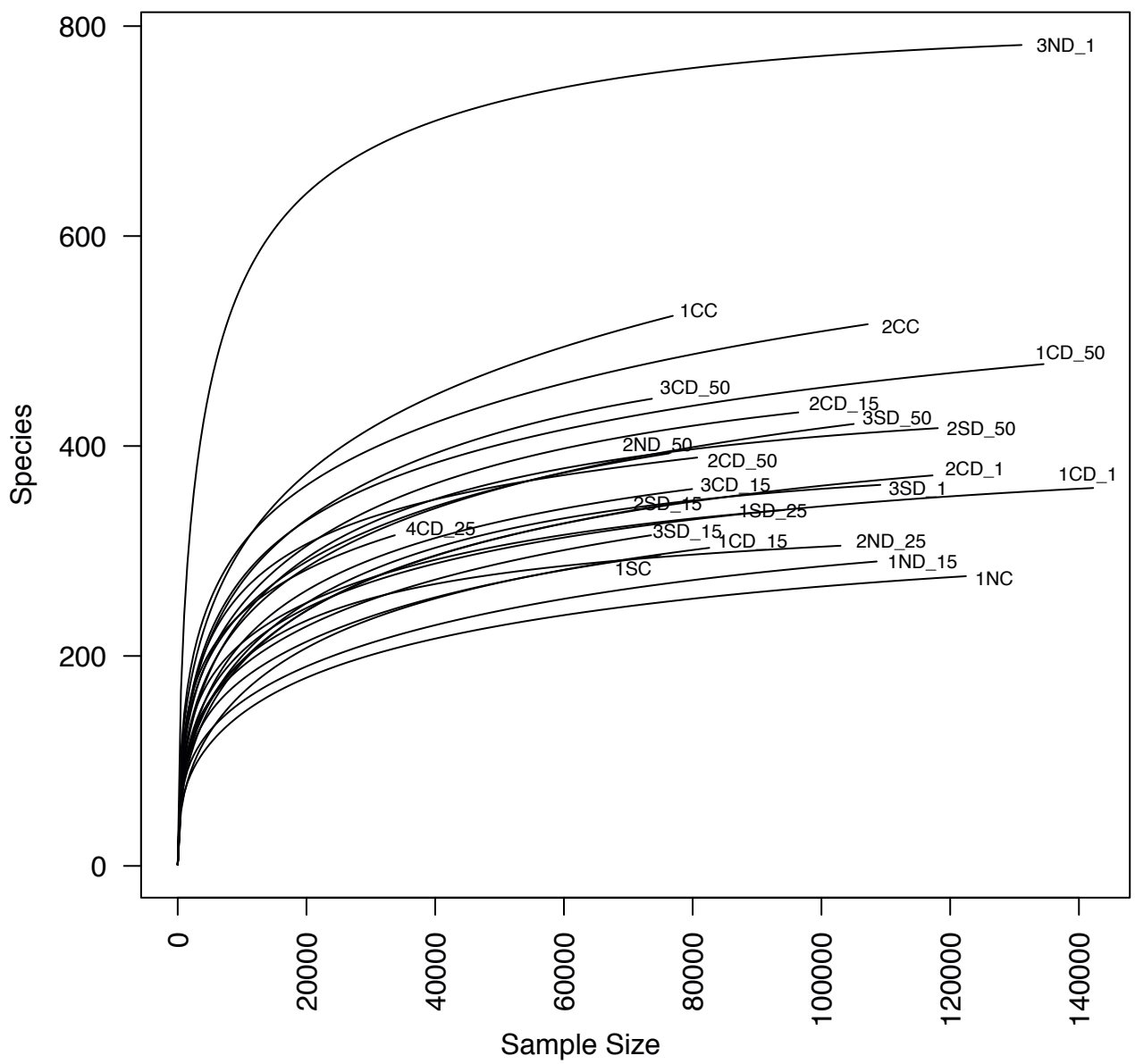

Supplement: Supplementary file 1 [file microorganisms-08-00543-s001.zip › Suppl_rev/S_1_Rarefaction.pdf]

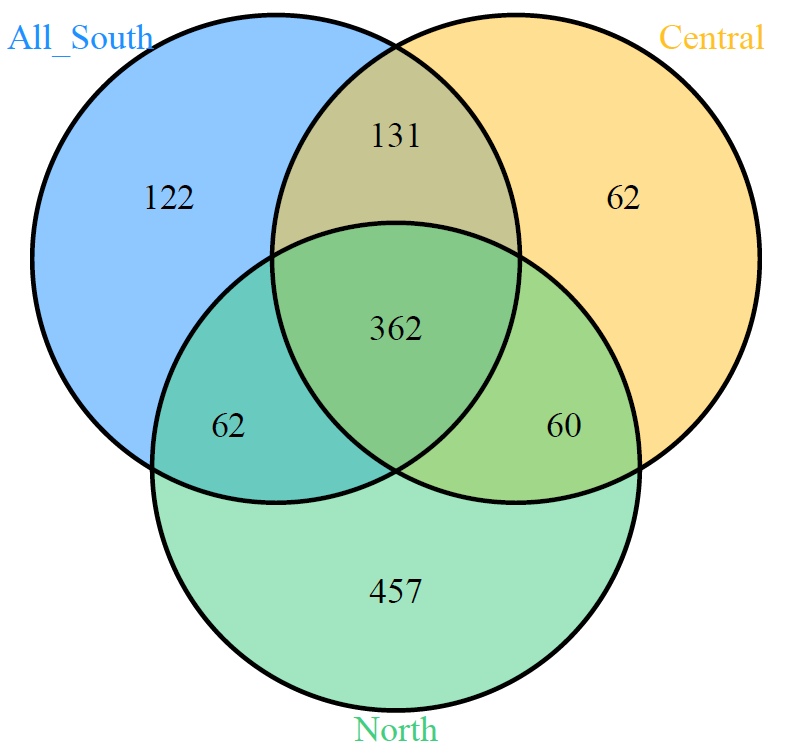

Supplement: Supplementary file 1 [file microorganisms-08-00543-s001.zip › Suppl_rev/S_12_Vien_basins.jpg]

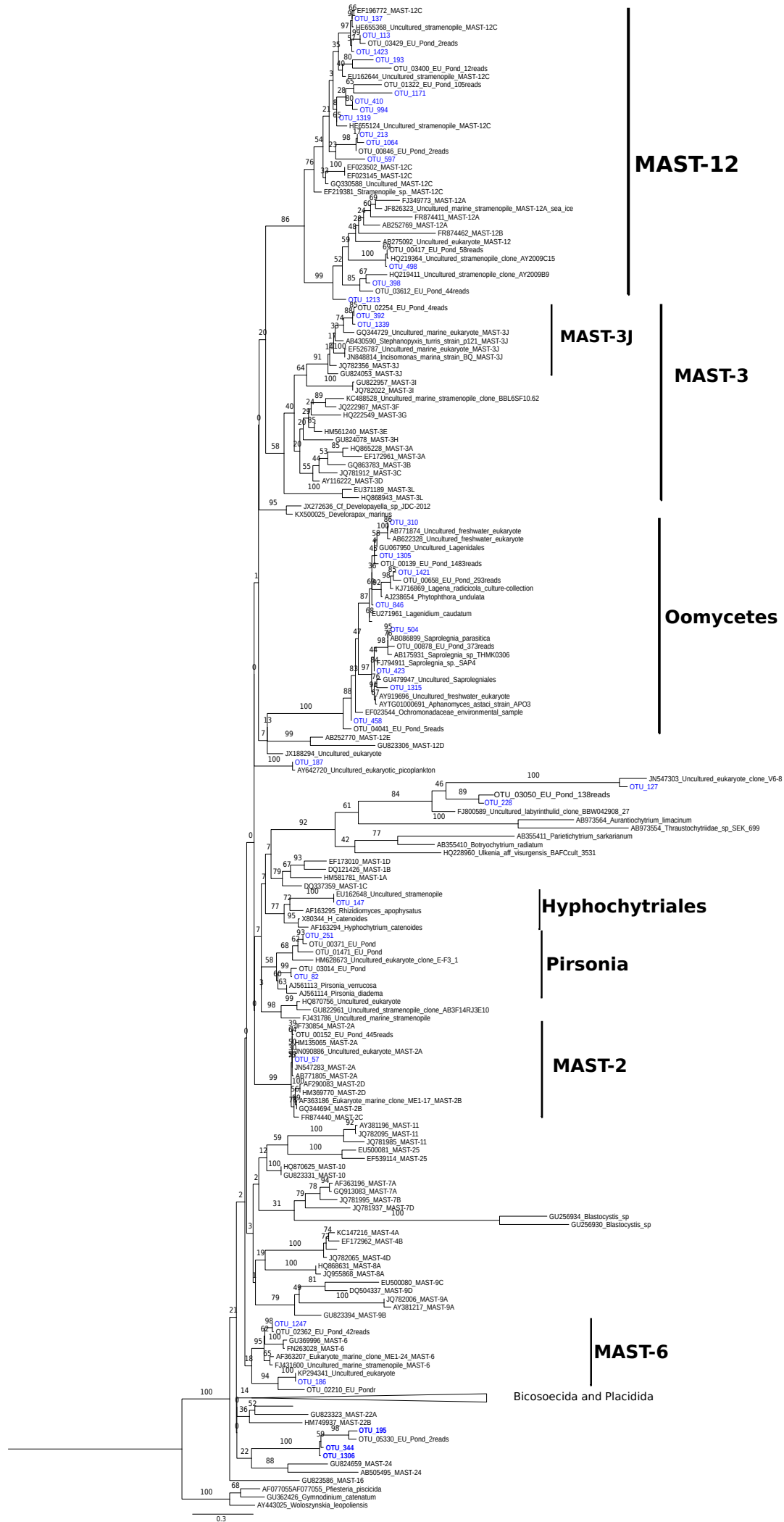

Supplement: Supplementary file 1 [file microorganisms-08-00543-s001.zip › Suppl_rev/S_4_other_Stramenopiles.pdf]
