# Supplementary material for: Tracing the Origin of Planktonic Protists in an Ancient Lake
Source: Microorganisms. 2020 Apr 9;8(4):543. doi: 10.3390/microorganisms8040543 (PMC7232311; doi:10.3390/microorganisms8040543)
Supplement: Supplementary file 1 [file S_10_opisthokonta_.pdf › Suppl_rev/S_10_opisthokonta_.pdf]

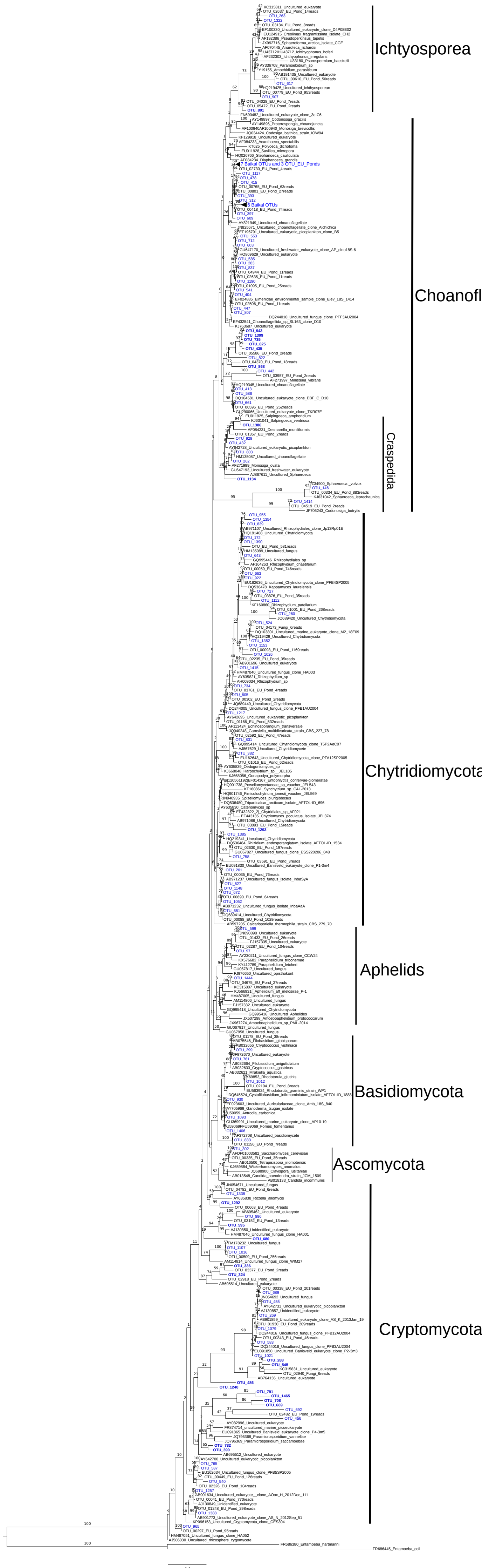
Ichtyosporea
Choanoflagellida
Craspedida
Chytridiomycota
Aphelids
Basidiomycota
Ascomycota
Cryptomycota
0.6
