# Supplementary material for: Tracing the Origin of Planktonic Protists in an Ancient Lake
Source: Microorganisms. 2020 Apr 9;8(4):543. doi: 10.3390/microorganisms8040543 (PMC7232311; doi:10.3390/microorganisms8040543)
Supplement: Supplementary file 1 [file microorganisms-08-00543-s001.zip › Suppl_rev/Supplement_description.doc]

Supplement_Fig. 1. Rarefaction curves of all samples.

Supplement_Fig. 2. Maximum Likelihood phylogenetic tree based on Baikal OTUs (in blue) and 18S RNA genes of protists from Ochrophyta obtained from databases.

Supplement_Fig. 3. Maximum Likelihood phylogenetic tree based on Baikal OTUs (in blue) and 18S RNA genes of protists from Alveolata (excluded ciliates) obtained from databases. Baikal OTUs, which has <90% similarity to other known sequences, are in bold.

Supplement_Fig. 4. Maximum Likelihood phylogenetic tree based on Baikal OTUs (in blue) and 18S RNA genes of protists from Stramenopiles (excluded Ochrophyta and Bicosoecida) obtained from databases. Baikal OTUs, which has <90% similarity to other known sequences, are in bold.

Supplement_Fig. 5. Maximum Likelihood phylogenetic tree based on Baikal OTUs (in blue) and 18S RNA genes of protists from Rhizaria obtained from databases.Baikal OTUs, which has <90% similarity to other known sequences, are in bold.

Supplement_Fig. 6. Maximum Likelihood phylogenetic tree based on Baikal OTUs (in blue) and 18S RNA genes of ciliates obtained from databases. Baikal OTUs, which has <90% similarity to other known sequences, are in bold.

Supplement_Fig. 7. Maximum Likelihood phylogenetic tree based on Baikal OTUs (in blue) and 18S RNA genes of protists from Archaeplastida obtained from databases. Baikal OTU, which has <90% similarity to other known sequences, is in bold.

Supplement_Fig. 8. Maximum Likelihood phylogenetic tree based on Baikal OTUs (in blue) and 18S RNA genes of protists from Hacrobia obtained from databases.

Supplement_Fig. 9. Maximum Likelihood phylogenetic tree based on Baikal OTUs (in blue) and 18S RNA genes of protists from Bicosoecida obtained from databases. Potential species flocks are marked by black ( OTUs difference was > 3%) and red (OTUs difference was < 3%) lines. Baikal OTUs, which has <90% similarity to other known sequences, are in bold.

Supplement_Fig. 10. Maximum Likelihood phylogenetic trees based on Baikal OTUs (in blue) and 18S RNA genes of protists from Opisthokonta obtained from databases. Baikal OTUs, which has <90% similarity to other known sequences, are in bold.

Supplement_Fig. 11. Maximum Likelihood phylogenetic trees based on Baikal OTUs (in blue) and 18S RNA genes of Chrysophyceae obtained from databases. Baikal OTUs, which has <90% similarity to other known sequences, are in bold.

Supplement_Fig. 12. Venn diagram showing the number of OTUs shared by Lake Baikal’s basins or specific to some of them.
